# Safer Conception Needs for HIV Prevention among Female Sex Workers in Burkina Faso and Togo

**DOI:** 10.1155/2014/296245

**Published:** 2014-10-23

**Authors:** Sheree R. Schwartz, Erin Papworth, Odette Ky-Zerbo, Simplice Anato, Ashley Grosso, Henri Gautier Ouedraogo, Sosthenes Ketende, Vincent Palokinam Pitche, Stefan Baral

**Affiliations:** ^1^Center for Public Health and Human Rights, Department of Epidemiology, Johns Hopkins Bloomberg School of Public Health, 615 North Wolfe Street, E7138, Baltimore, MD 21205, USA; ^2^Programme d'Appui au Monde Associatif et Communautaire (PAMAC), 11 BP 1023 Ouagadougou, Burkina Faso; ^3^Arc en Ciel, 80295 Lomé, Togo; ^4^Institut de Recherche en Sciences de la Santé (IRSS/CNRST), 03 BP 7192 Ouagadougou, Burkina Faso; ^5^Conseil National de Lutte contre le SIDA du Togo (National AIDS Council), Faculté des Sciences de la Santé, Université de Lomé, 01 BP 2237 Lomé, Togo

## Abstract

*Background*. Reproductive health programming for female sex workers (FSW) may include contraceptive services but rarely addresses safer pregnancy planning. *Methods*. Adult FSW were enrolled into a cross-sectional study across four sites in Burkina Faso and Togo using respondent-driven sampling. Sociobehavioral questionnaires and HIV counseling and testing were administered. Sample statistics and engagement in HIV treatment were described and compared using Chi-squared statistics. *Results*. 1,349 reproductive-aged FSW were enrolled from January to July 2013. Overall, 267 FSW (19.8%) were currently trying to conceive. FSW trying to conceive were more likely to test positive for HIV at enrollment as compared to women not trying to become pregnant (24.5% versus 17.7%, *P* < 0.01); however awareness of HIV status was similar across groups. Among FSW trying to conceive, 79.0% (211/267) had previously received HIV testing, yet only 33.8% (23/68) of HIV-infected FSW reported a previous HIV diagnosis. Overall 25.0% (17/68) of HIV-infected FSW trying to conceive were on antiretroviral therapy. *Conclusion*. FSW frequently desire children. However engagement in the HIV prevention and treatment cascade among FSW trying to conceive is poor potentiating periconception transmission risks to partners and infants. Programs to facilitate earlier HIV diagnosis for FSW and safer conception counseling are needed as components of effective combination HIV prevention services.

## 1. Introduction

Reproductive health programming for female sex workers (FSW) has traditionally emphasized contraception, including condom use for both pregnancy and HIV prevention, and screening and treatment of sexually transmitted infections [1–4]. While these are critical components of reproductive health programming, fertility is of great importance in Sub-Saharan Africa [5–7], and there is growing evidence that FSW, like other women, desire children [8–10]. Although the need for comprehensive reproductive healthcare for FSW has been raised [11], to date limited attention has been given to safer pregnancy planning for FSW.

There has been increased awareness of the safer conception needs of people living with HIV in general [12–15]; however service provision is scarce in Sub-Saharan Africa [16], and safer conception counseling received by HIV-affected couples has been limited [12, 17, 18]. Safer conception approaches employ risk-reduction strategies for HIV-affected couples trying to conceive, such as biomedical approaches to ensure viral suppression of HIV-infected partner(s) and behavioral strategies to reduce and efficiently time the number of unprotected vaginal sex acts [15]. As FSW are disproportionately affected by HIV, safer conception approaches are particularly relevant for FSW trying to become pregnant, in order to minimize periconception HIV infection and superinfection risks among FSW, to minimize onward transmission risks to partners of FSW living with HIV and to reduce risks to infants [19].

Several barriers may prevent FSW in West and Central Africa from being reached by safer conception counseling. HIV prevalence across the region has been estimated at nearly 35% among FSW [20], a burden far exceeding that of other reproductive-aged women in the region [21]; however safer conception knowledge and services are likely more scarce than those available to women in more generalized epidemics. In addition to poor availability of safer conception services in general, knowledge of HIV status among FSW may be low and engagement in care limited [22, 23]. Furthermore, among FSW engaged in HIV care, women who sell sex may not feel comfortable discussing reproductive desires with healthcare providers out of fear that providers would not support their reproductive choice or provide preconception care based on their occupation or HIV status [24].

Limited evidence is available about FSW trying to conceive, including whether HIV prevention strategies are being adopted to reduce risk of HIV acquisition or transmission to partners and infants in the periconception period. For FSW trying to conceive, engagement in the HIV treatment cascade—including HIV testing and awareness of HIV status, linkage to HIV care and/or prevention of mother-to-child transmission (PMTCT) services, antiretroviral therapy (ART) initiation, and adherence to treatment (viral suppression) [25]—is critical for the optimization of health outcomes for FSW, partners, and infants. The objective of this analysis is to assess fertility intentions and HIV among FSW, as well as engagement in the HIV prevention, treatment, and care cascade among FSW trying to conceive in two West African countries—Burkina Faso and Togo.

## 2. Methods

### 2.1. Recruitment Methods and Study Population

Studies with key populations (populations most at risk of HIV infection), including FSW and men who have sex with men, were conducted in four sites including Kara and Lome in Togo and Ouagadougou and Bobo-Dioulasso in Burkina Faso. FSW were recruited into the research using respondent-driven sampling (RDS). Methodology has been described in detail elsewhere [26], but, briefly, RDS is chain-referral method used to recruit hard-to-reach populations. A select number of “seeds” who met study eligibility criteria and were well connected within the FSW community were purposively identified in coordination with local stakeholders from the sex worker community and invited to participate in the study. Seed selection took into account seed characteristics in order to ensure that a range of ages, years engaged in sex work, countries of origin (local versus foreign-born FSW), education, marital status, and languages spoken were represented. Seeds were released gradually over time to ensure steady enrollment. Each consenting seed (*n* = 20) participated in all study activities and was then given three coupons to invite other eligible FSW. The invitees returned the coupons to the study site and, if eligible, were also enrolled and completed study procedures after which they also became recruiters and were given three coupons to invite other known acquaintances in their network to participate. This process continued until the sample size was met for each site. Methods for recruiting and enrolling participants were the same across study sites.

Participants were reimbursed for average local transport costs to the study site and the equivalent of a small meal, 2,000 CFA in Burkina Faso (approximately USD 4) and 5,000 CFA (approximately USD 10) in Togo. Secondary reimbursements to compensate for time and expenses used recruiting other FSW into the study were also provided for each eligible participant (up to three) that the participant successfully recruited. Secondary reimbursements were 1,500 CFA (approximately USD 3) per eligible recruited FSW in Burkina Faso and 3,000 CFA (approximately 6 USD) in Togo. Reimbursements varied across countries based on differences in average local transport costs to study sites and food costs. All participants also received free male condoms and HIV prevention and treatment information.

Study procedures for all enrolled participants included a cross-sectional sociobehavioral questionnaire and HIV counseling and rapid testing. Questionnaires were administered by experienced quantitative interviewers who had completed training in research ethics and study procedures. HIV counseling and rapid testing was done in accordance with the national guidelines in each country. HIV counselors administered the pre- and posttest counseling, while a laboratory technician collected blood samples from the participants and processed the HIV tests. The HIV testing algorithm in both countries was sequential. In Burkina Faso,* Determine HIV 1/2 Ag/Ab Combo Rapid Test* was used and all positive results were confirmed with HIV Bispot ImmunoComb 11. Discordant test results were confirmed using ImmunoComb II HIV 1&2 CombFirm. In Togo, the* Determine HIV 1/2 Ag/Ab Combo Rapid Test* was also used; however sequential testing of positive samples was conducted using First Response HIV test 1–20 cards (PMC Medical). Western blots were used for any discrepant results. All participants received pretest counseling and were counseled of the benefits of knowing one's status however participants could choose whether or not to receive their HIV test results. Referrals to treatment and care were made for anyone receiving a confirmed positive HIV test result not already engaged in either pre-ART care or treatment.

FSW were eligible to participate in the study if they were ≥18 years old and born female, reported that the majority of their income within the past 12 months was obtained from sex work, presented a valid RDS coupon, and had not previously participated in the study. Informed consent was administered in French or the locally spoken language at each location. Study participation was anonymous, and all study-related procedures were conducted in private rooms at secure study sites. As the results focus on fertility intentions and the need for preconception care among reproductive-aged FSW, women >49 years of age were excluded from the present analysis.

### 2.2. Measures

Assessment of fertility intentions, particularly the indicator “trying to conceive,” refers to FSW who indicated that they are currently trying to become pregnant. For this analysis we considered risk taking behaviors to be behaviors which may increase risk of horizontal or vertical transmission of HIV in the periconception period, such as having untreated sexually transmitted infections (STIs), unprotected vaginal or anal sex with a nonpaying partner, and having multiple nonpaying partners. Conversely, risk mitigating behaviors among women trying to conceive were behaviors which may decrease periconception HIV transmission risk, such as engaging in HIV testing, 100% condom use with clients, and talking to nonpaying partners and clients about HIV. Awareness of HIV status and use of ART were self-reported.

### 2.3. Statistical Analysis

This is a secondary data analysis of data collected to estimate HIV prevalence across sites as well as engagement in HIV prevention and treatment activities. Secondary objectives of the study included improved understanding of risks for HIV infection, reproductive health, and human rights violations among FSW.

Descriptive statistics for the overall sample of women of reproductive age are provided. Throughout the analyses we do not present RDS-adjusted estimates, as RDS adjustment is not appropriate when sample populations across study sites are combined [27]. Among women trying to conceive, descriptive statistics of HIV prevention and risk behaviors were compared using Chi-squared statistics between women with reactive and nonreactive HIV test results at enrollment. Engagement in the HIV treatment cascade was further illustrated among women trying to conceive. HIV status and engagement in steps of the treatment cascade were further compared between women who were and who were not trying to conceive using Chi-squared statistics.

## 3. Results

Between January and July 2013, 1,380 FSW were enrolled at study sites in Burkina Faso and Togo. Thirty-one women were excluded from this analysis as they were above reproductive age (>49 years), leaving the analysis population to 1,349. The median age was 25 years (interquartile range (IQR) 22–32). Crude HIV prevalence among women enrolled was 19.2% (95% confidence interval (CI) 17.1–21.3) and ranged from 8.5% (95% CI 5.5–11.4) in Ouagadougou to 31.9% (95% CI 26.9–36.8) in Bobo-Dioulasso.  Table 1 presents the characteristics of the women included in the analysis. The majority of women had never been married (55.7%) and had at least one child (70.4%). The distribution of years engaged in sex work was roughly split between those newer to sex work (0–2 years of experience), those with 2–5 years of experience, and FSW engaging in sex work for more than 5 years. Overall, 267 out of the 1,349 FSW (19.8%) reported that they were trying to become pregnant at the time of the study. Long-acting reversible methods (LARC) of contraception were used by 24.8% of women, including 24 women (9.0%) currently trying to conceive.

### 3.1. HIV-Related Risks in the Periconception Period

**Figure 1:**
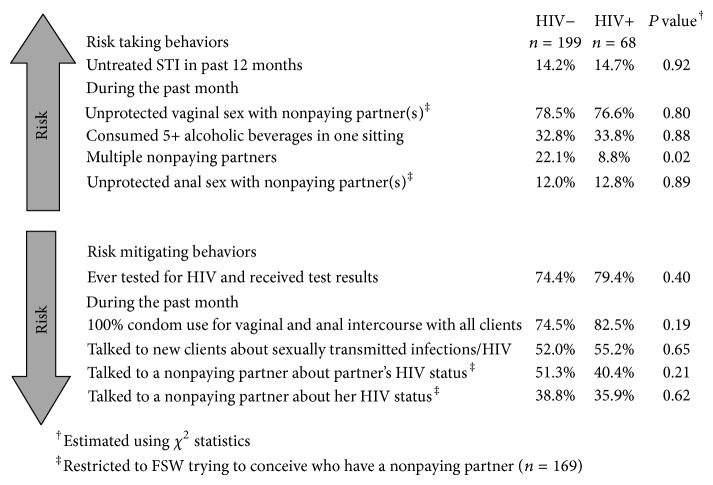
HIV prevention and risk behaviors among female sex workers trying to conceive (*n* = 267).

FSW trying to conceive trended to more frequently report unprotected vaginal sex with nonpaying partner(s) in the past month as compared to FSW who also had nonpaying partner(s) but were not trying to conceive (77.9% versus 70.6%, resp., *P* = 0.06). Limited to the subset of FSW trying to conceive, Figure 1 further demonstrates the frequency of reported behaviors that elevate or mitigate HIV-related risks in the periconception period. Among FSW trying to conceive, the proportion of women reporting unprotected vaginal sex acts with nonpaying partners was similar between women living with and without HIV (76.6% versus 78.5%, *P* = 0.92). Around 12% of women reported unprotected anal sex with their nonpaying partners in the past month as well. Overall in the Chi-squared analyses assessing periconception behaviors that increase HIV transmission, reporting multiple nonpaying partners was the only risk behavior associated with HIV infection status among women trying to conceive. FSW living with HIV were less likely to report multiple nonpaying partners as compared to HIV-uninfected FSW. This may suggest that FSW known to be living with HIV reduce the number of their nonpaying partners to minimize onward transmission risk, while HIV-uninfected FSW may take greater personal risks in order to become pregnant. In terms of behaviors known to mitigate risk, HIV-infected and uninfected women were similar. Just over one-third of women had talked to their sexual partners in the past month about their HIV infection status, and slightly more women had talked to their nonpaying partners about his HIV infection status. History of ever testing for HIV was comparable between HIV-infected (79.4%) and -uninfected (74.4%, *P* = 0.40) women who were trying to conceive, as was consistent condom use during sex acts with clients (82.5% and 74.5%, resp., *P* = 0.19).

### 3.2. Engagement in HIV Care among FSW Trying to Conceive

**Figure 2:**
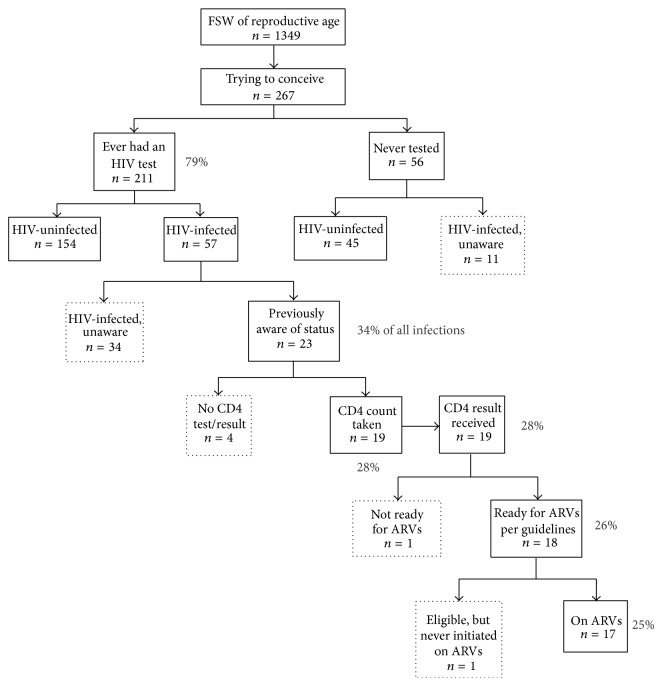
Engagement in the HIV treatment cascade among female sex workers trying to conceive.

Engagement in the HIV treatment cascade among FSW trying to conceive is illustrated in Figure 2. Solid boxes in the cascade diagram represent women retained in care, whereas dotted lines represent individuals who have fallen out of care at various points across the continuum. Among women trying to conceive, 79.0% (*n* = 211) reported ever being tested for HIV. Of the 56 women never tested for HIV, 19.6% were living with HIV and reportedly unaware of their status. Twenty-seven percent of the 211 women who had previously tested were also living with HIV and reportedly unaware of their status. In total, 25.5% (68 of 267) FSW trying to conceive were living with HIV, and 66.2% (45/68) of HIV-infected women reported no previous HIV diagnosis. The majority (82.6%, *n* = 19/23) of women aware of their HIV status reported that they had previously had a CD4 count taken, all of whom received the results and 94.7% of whom were eligible for treatment per the national guidelines. All but one FSW trying to conceive who had received CD4 testing and was ART-eligible was on treatment; however, ART coverage among all HIV-infected FSW trying to conceive was 25.0% (17/68).

There were differences in engagement in the care continuum between women in Togo and Burkina Faso. In Togo 23.7% of HIV-infected FSW trying to conceive reported awareness of their status, as compared to 51.9% in Burkina Faso (*P* = 0.02). ART coverage among HIV-infected women was 13.2% in Togo and 44.4% in Burkina Faso (*P* < 0.01).

In terms of comparisons in engagement in HIV care between FSW trying to become pregnant and those not trying to conceive, HIV testing history, reported awareness of HIV infection status, and reported HIV treatment uptake were comparable between the two groups (Table 2). Women trying to conceive were more likely to be living with HIV (24.5%) as compared to women not trying to become pregnant (17.7%, *P* < 0.01). The trend of higher fertility intentions among FSW living with HIV versus HIV-uninfected FSW was seen across age strata (18–24 years, 25–35 years, and ≥36 years); however this relationship was only statistically significant among women aged 25–35 years (results not shown). In terms of other demographics, engagement in ART treatment among FSW living with HIV and trying to conceive was similar among married/cohabitating FSW and nonmarried FSW (36.4% versus 24.1%, resp., *P* = 0.71), as well as among women who were already mothers versus nonmothers (26.8% versus 25.0%, *P* = 0.87).

## 4. Discussion

A substantial proportion of FSW in the sample were trying to become pregnant and both FSW living with HIV and HIV-uninfected FSW were facing periconception HIV transmission risks. HIV-uninfected women trying to conceive were engaged in frequent unprotected sex with their nonpaying partners, increasing the potential for exposure to HIV. Furthermore, fertility intentions were higher among women living with HIV as compared to HIV-uninfected FSW. Within the subset of women trying to conceive, one-fourth were living with HIV, but two-thirds of HIV-infected women reported being unaware that they were living with HIV. Among the subset of women aware of their HIV infection status, there was high reported engagement in HIV care, including CD4 staging and ART initiation; however large gaps in infection diagnosis resulted in low overall ART coverage among HIV-infected FSW trying to conceive. Treatment coverage gaps were evident in both married/cohabitating and nonmarried women, as well as FSW who were already mothers and nonmothers.

Risks to FSW and their partners in the periconception period reinforce the importance of earlier HIV infection diagnosis and treatment as prevention for serodiscordant partnerships independent of CD4 count per World Health Organization guidelines [28]. Low ART coverage among HIV-infected FSW trying to conceive may result in high risks for HIV transmission to HIV-uninfected male partners and to infants [29]. This may be particularly true among the FSW sampled, as the majority of FSW trying to conceive appeared to be unaware of their status and thus less likely to utilize other HIV prevention measures while trying to conceive, such as unprotected sex limited to the periovulatory period, self-insemination, or preexposure prophylaxis by the male partner to reduce transmission risks [16]. Equally, FSW living with HIV but unaware of their status may be less motivated to attend antenatal care and engage in PMTCT programs during early pregnancy. Furthermore, frequent occupational exposure through sex work to HIV and the possibility of HIV superinfection during the periconception period may result in higher viral load among FSW and greater transmission risks to HIV-uninfected partners compared to other serodiscordant relationships [30].

Women trying to conceive in our analysis had comparable ART coverage to women who were not trying to become pregnant. However, women trying to conceive were more likely to be living with HIV than women who were not trying. As this study was cross-sectional, causality cannot be determined; however there was no evidence in our study that living with HIV caused women to be more likely to want to conceive. Many FSW living with HIV in our study did not report a previous diagnosis, and prior HIV diagnosis (unlike actual HIV-infection status) was not associated with fertility intentions. Other studies with HIV-infected African women of reproductive age have reported that women known to be living with HIV have lower fertility intentions than HIV-uninfected women [31, 32]. Alternatively, our findings may indicate that attempted conception may be increasing risk for HIV infection. Outside of self-insemination and more advanced reproductive technologies, unprotected sex is required to conceive; thus HIV transmission risks may be higher among women trying to conceive further reinforcing the importance of treatment in serodiscordant partnerships and low-cost strategies to conceive while minimizing potential exposure to HIV. Indeed, among both HIV-infected and HIV-uninfected FSW trying to conceive, unprotected sex with nonpaying partners was common, and communication with partners about HIV was low. Other studies have also documented frequent sex without condoms among women trying to become pregnant [33].

We are not aware of any published HIV prevention studies which have provided FSW with safer conception services to minimize risk for HIV acquisition and transmission. While there is increasing awareness that FSW may have fertility desires [8, 10], this appears not to have translated into safer conception service provision. Within the Sub-Saharan African context more generally, safer conception guidelines have been published [15], but services for couples affected by HIV remain largely unavailable outside of individual providers who may offer counseling. Even in the cases in which services have been offered, generating demand takes time [34], as many individuals may want to become pregnant but may not recognize the related need for engagement in HIV care, including safer conception services.

FSW living with HIV are harder to reach with preconception care than women who do not sell sex, due to low existing engagement in HIV care among FSW [22, 23], and perceptions or experiences of intersecting stigma related to their reproductive goals as sex workers and women living with HIV [35–38]. Given that many FSW are trying to conceive, safer conception counseling services are required, alongside efforts to reduce stigma and other barriers to engaging in HIV testing and care programs.

In mixed HIV epidemics like Burkina Faso or Togo, health care providers may be less aware of safer conception methods and may be less equipped to counsel FSW affected by HIV about how to conceive safely. Development of improved clinical and cultural competencies to counsel FSW and their partners about fertility and low-cost methods for safer conception will be required to ensure that messaging is clear and appropriate. These results suggest the need for this training to be integrated into both clinical HIV and family planning curricula for health care providers treating FSW.

These results represent a sample of women in two West African countries recruited using nonrandom sampling methods and thus may not be generalizable to other FSW within the study countries or elsewhere. Our sample includes 1,349 FSW in Burkina Faso and Togo who may be very different from other FSW not reached through the RDS sampling methods. Despite this limitation, truly random samples with FSW are difficult to achieve given the fact that there is no sampling frame for FSW and venue-based methods for recruitment are also limited by the study team's ability to enumerate and engage with all sex work venues. Peer recruitment methods represent a way of reaching women that may otherwise not be found as well as FSW not already engaged in existing FSW programs or services. Thus, despite the limitations of a nonrandom sample, our study sample demonstrates the potential magnitude of safer conception needs. Furthermore, although both fertility intentions and engagement in the HIV treatment cascade may differ among FSW in other Sub-Saharan African countries with more generalized epidemics, HIV prevalence among FSW is also higher in those settings and thus there is likely a need for safer conception services in other settings [19]. Causal attribution of HIV transmission risks related to attempted conception cannot be made from these cross-sectional data. Information about disclosure to partners and the HIV status of FSW's partners with whom they were trying to conceive was not collected and would strengthen our understanding of HIV transmission risks in this context. Similarly, we did not collect information on safer conception counseling received or strategies employed by FSW, nor do we have objective confirmation of adherence to ART and achievement of viral suppression among women trying to conceive who are living with HIV. However based on self-reported treatment data, the best case scenario of viral suppression among FSW living with HIV and trying to conceive in this sample is approximately 25% (17/68) if all HIV-infected FSW on treatment were virally suppressed.

## 5. Conclusion

The HIV transmission risks among individuals and couples trying to conceive in the context of HIV are likely not evenly distributed in the population. High fertility intentions and unprotected vaginal sex in order to achieve conception increase the HIV acquisition risks experienced by FSW. Furthermore, low engagement in repeat HIV testing and care among FSW trying to conceive increases the HIV transmission risks to their partners and children. Improving access to and uptake of HIV prevention and treatment services for FSW, including safer conception counseling, could help to optimize HIV prevention goals and support the reproductive rights of FSW.

## Figures and Tables

**Table 1:** Characteristics of female sex workers of reproductive age in key populations studies in Togo and Burkina Faso.

Characteristics (*n* = 1349)	Sample estimates	
	*n*	%
Study site		
Kara, Togo	327	24.3
Lome, Togo	340	25.2
Ouagadougou, Burkina Faso	343	25.4
Bobo-Dioulasso, Burkina Faso	339	25.1
Country of birth		
Togo	578	42.8
Burkina Faso	526	39.0
Ghana	92	6.8
Ivory Coast	87	6.5
Others	66	4.9
Age, years		
18–24	603	44.7
25–35	520	38.5
36–49	226	16.8
HIV prevalence	259	19.2
Highest education completed^†^		
Less than primary	558	41.5
Primary	274	20.4
Secondary (middle school)	391	29.1
High school or beyond	122	9.0
Income past month, USD		
Less than $150	438	32.5
$151–299	494	36.6
$300 or more	417	30.9
Location where sex work clients are met^‡^		
Bar/club	1014	75.2
Brothel	431	31.9
Street/park	795	58.9
Hotel/guest house	482	35.7
Private home	213	15.8
Marital status		
Married/cohabitating	117	8.7
Never married	752	55.7
Divorced/separated/widowed	480	35.6
Current nonpaying partner		
Yes	968	71.8
No	381	28.2
Number of biological children		
None	399	29.6
One child	430	31.9
Two or more children	520	38.5
Years selling sex		
≤2 years	429	31.8
>2–5 years	453	33.6
>5	467	34.6
Number of clients in the past month^§^		
0–10	514	38.1
11–20	355	26.3
>20	480	35.6

^†^Four individuals were missing education data (*n* = 1345). ^‡^The column percent exceeds 100% as participants may work in multiple locations. ^§^22/1349 (1.6%) FSW reported zero clients in the past month. All participants met study criteria, but some may not have engaged in sex work in the past 30 days due to choice, illness, travel, family obligations, or other reasons.

**Table 2:** HIV status and engagement in care by fertility intentions of female sex workers sampled in two cities in Burkina Faso and two cities in Togo (*n* = 1,349).

	FSW not trying to conceive	FSW trying to conceive	*P* value
	*n* = 1,082	*n* = 267	
Ever tested for HIV prior to study enrollment, *n* (%)	886 (82.0%)	211 (79.0%)	0.26
HIV status per biological testing at enrollment, *n* (%)			
HIV-uninfected	891 (82.3%)	199 (74.5%)	<0.01
HIV-infected	191 (17.7%)	68 (24.5%)	
Reported previous awareness of HIV status, *n* (%)^†^	74 (38.7%)	23 (33.8%)	0.72
Reported antiretroviral therapy (ART) use, *n* (%)^†^	63 (34.1%)	17 (26.2%)	0.24

^†^Restricted to HIV-infected FSW with self-reported diagnosis and ART data (*n* = 250).
